# John Nathan Crouse, D. D. S.

**Published:** 1914-02-15

**Authors:** 


### OBITUARY.

John Nathan Crouse, D. D. S.:—Dr. Crouse the organizer and president of the Dental Protective Association died at his home in Chicago, January 16th, in his seventy-second year of age. Dr. Crouse was born near Downington, Chester Co., Pa., September 15th, 1842. He prepared for college in the Mt. Carroll, Ill., Seminary, 1859 to 1862. He graduated from the Pennsylvania College of Dental Surgery in the spring of 1867. He began practice in Mt. Carroll, but removed to Chicago in 1868, where he has been continuously in active practice up to the time of his death. He has always taken an active interest in professional matters. He was the last of the original or charter members of the Illinois State Dental Society. He was always an active participant in the proceedings of the State and Chicago Dental Societies. For many years he was a member of the executive committee of the American Dental Association, and through this organization he rendered much valuable service to the profession at large. Probably his greatest service, and the one which will be best known for all time was the work of organizing the Dental Protective Association for the protection of the profession from unfair patent sharks. He spent much time and money of his own in his efforts to prevent owners of crown and bridge patents from collecting royalties from the profession, and finally succeeded in having these patents declared invalid by the courts.

He tried to organize a dental supply house to be owned and controlled by members of the Dental Protective Association, but the profession did not take to the proposition and Dr. Crouse had to assume the burden of its conduct. It is said that this venture absorbed all his money and encroached upon his time and became a serious burden to him in his last days.

Together with his son, D. H. Crouse, he established the *Dental Digest* which they successfully conducted until the death of his son, January 3rd, 1906. After two years, Dr. Crouse sold the *Digest* to The Dentists' Supply Co., of New York, which concern has since published this journal with Dr. Geo. W. Clapp as editor.

Dr. Crouse had a fine practice among the most prosperous people of Chicago, and had he confined his attention to his private practice he would have died a wealthy man. He was an optomist and had great visions of the future of his profession, and he was glad to make every sacrifice of time and money to do his part in promoting professional advancement of every kind. He was one of the organizers and continued for many years an advisor of the Chicago Dental

College. It is hard to think of a mans being dead who has always been so active in our professional life. His last service to the profession was to make an arrangement with Dr. W. H. Taggart whereby the members of the Dental Profession could secure for a very small compensation the right to use all the patents owned by Dr. Taggart on the methods and machine for casting gold inlays. The profession of the whole country will learn of his death with sorrow. He was a kind friend, and we shall cherish in pleasant memory our fellowship.

